# Effects of ganciclovir combined with recombinant human interferon-α on clinical efficacy and immune function in children with infectious mononucleosis

**DOI:** 10.12669/pjms.40.7.8705

**Published:** 2024-08

**Authors:** Ling Sun, Jing Bi, Weina Zhen, Meiying Wang, Haobin Song

**Affiliations:** 1 Ling Sun, Department of Otology, Baoding Key Laboratory of Clinical Research on Children’s Respiratory and Digestive Diseases, Department of Ophthalmology and Otorhinolaryngology, Baoding, Hebei, China. Department of Infectious Diseases, Beijing Children’s Hospital Affiliated to Capital Medical University Baoding Hospital, Baoding 071000, Hebei, P.R. China; 2 Jing Bi, Department of Infectious Diseases, Beijing Children’s Hospital Affiliated to Capital Medical University Baoding Hospital, Baoding 071000, Hebei, P.R. China, Baoding Accurate Diagnosis and Treatment Laboratory of Children’s Infectious Diseases, Baoding 071000, Hebei, P.R. China; 3 Weina Zhen, Department of Infectious Diseases, Beijing Children’s Hospital Affiliated to Capital Medical University Baoding Hospital, Baoding 071000, Hebei, P.R. China, Baoding Accurate Diagnosis and Treatment Laboratory of Children’s Infectious Diseases, Baoding 071000, Hebei, P.R. China; 4 Meiying Wang, Department of Laboratory, Baoding Hospital of Traditional Chinese Medicine, Baoding 071000, Hebei, P.R. China; 5 Haobin Song, Department of Laboratory, Department of Infectious Diseases, Beijing Children’s Hospital Affiliated to Capital Medical University Baoding Hospital, Baoding 071000, Hebei, P.R. China

**Keywords:** Ganciclovir, Recombinant human interferon-α, Children, Infectious mononucleosis, Immune function, Treatment

## Abstract

**Objective:**

To evaluate the effects of ganciclovir combined with recombinant human interferon on clinical efficacy and immune function of children with infectious mononucleosis(IM).

**Methods:**

This was a retrospective study. Children (n=120) with IM hospitalized in Beijing Children’s Hospital Affiliated to Capital Medical University Baoding Hospital from January 2020 to January 2022 were selected and randomly divided into study group and control group((n=60). Patients in the control group were treated with ganciclovir by intravenous infusion, and patients in the study group were given ganciclovir+recombinant human interferon-α1b. The time for eliminating clinical symptoms, the levels of inflammatory cytokines, immune function condition and T-lymphocyte subsets between the two groups were compared and analyzed.

**Results:**

After treatment, the time for body temperature returned to normal, time for recovery from cervical lymphadenopathy, time for recovery from hepatosplenomegaly and time for disappearance of angina and oral mucosal congestion in the study group were significantly shorter than those in the control group(p= 0.00); after treatment, the levels of TNF-a and IL-6 in the study group were significantly lower than those in the control group; the indexes of CD3^+^ and CD8^+^ in the study group were significantly lower than those in the control group; after treatment, the levels of CD4^+^ and CD4^+^/CD8^+^ in the study group were significantly higher than those in the control group.

**Conclusion:**

Ranciclovir combined with recombinant human interferon-α1b, rapid improvements of clinical symptoms, significantly decreased inflammatory cytokines, improved T-lymphocyte function and no significant increase in adverse drug reactions were found in children with IM.

## INTRODUCTION

Infectious mononucleosis (IM) was a common disease in the Department of Paediatrics, and more than 90% of IM was caused by Epstein-Barr virus (EBV).^1^ IM was mainly transmitted by close contact via the oral route, and was more likely to occur in preschool and school-age children.^2^ Specifically, the main clinical manifestations of IM were fever, angina, hepatosplenic lymphadenopathy^3^ and liver function damage.^4^ IM was characterized by diverse clinical manifestations and lack of specific clinical symptoms and signs in the early stage^5^, and IM was prone to causing damage to multiple organs and systems of child patients to a certain extent, thereby seriously affecting the growth and development of children.^6^

Ganciclovir, an antiviral drug, could achieve an antiviral effect by stopping the growth of viral DNA strand. However, the efficacy of single antiviral therapy in IM was still uncertain. At present, the quality of evidence-based literature was very poor. Most of the included studies were unclear or at high risk of bias, thus the effectiveness of the single treatment plan remained questionable.^7^ Kaul et al.^8^ proved that immunomodulators, as adjuvant therapy for IM, showed good clinical efficacy since the immunomodulators could improve cellular immune function, and had a synergistic effect with antiviral drugs. In the present study, a treatment plan of ganciclovir combined with recombinant human interferon was used for IM in children, to evaluate the effects of ganciclovir combined with recombinant human interferon-α1b on clinical efficacy and immune function of children with infectious mononucleosis(IM)

## METHODS

This was a retrospective study. A total of 120 cases of IM in Beijing Children’s Hospital Affiliated to Capital Medical University Baoding Hospital were selected from January 2020 to January 2022 and randomly divided into two groups (60 cases in each group). There was no significant difference in general data between the two groups(*p>* 0.05), and there was comparability between the two groups(Table-I).

**Table-I:** Comparison and analysis of general data of study group and control group (n = 60 per group) (*χ̅*±*S*).

Item	Study group	Control group	t/χ^2^	P
Age (year)	7.57 ± 2.76	7.89 ± 2.94	0.61	0.54
Male (n%)	32 (53.3%)	36 (60%)	0.54	0.46
Fever at first visit (%)	51 (85%)	53 (88%)	0.29	0.59
Lymphadenopathy (%)	40 (66.7%)	38 (63.3%)	0.15	0.70
Hepatosplenomegaly (%)	20 (33.3%)	22 (36.7%)	0.15	0.70
Course of disease before treatment course	3.78 ± 1.21	3.75 ± 1.07	0.14	0.89

### Ethical Approval

The study was approved by the Institutional Ethics Committee of Beijing Children’s Hospital Affiliated to Capital Medical University Baoding Hospital (No.: 2019-22; date: November 25, 2019), and written informed consent was obtained from all participants’ guardian.

### Inclusion criteria:

- Child patients meeting the diagnostic criteria of infectious mononucleosis(IM);^9^
- Infectious mononucleosis children aged 3-14 years old or below;
- Infectious mononucleosis children with complete clinical data, but without allergic reaction to drugs used in the study;
- Infectious mononucleosis children without other treatment given before admission;
- Infectious mononucleosis children with family members willing and able to cooperate to complete the study;

### Exclusion criteria:

- Patients combined with severe organic diseases of heart, liver and kidney or congenital diseases;
- Patients who were allergic to the drugs needed in the study;
- Patients with recent use of drugs affecting the study in the past one month, such as immunomodulators, hormones, etc.;
- Child patients combined with autoimmune diseases;
- Patients combined with other viral infections, such as cytomegalovirus infection, hepatitis virus infection, etc.;
- Patients with mental system disease, or patient who were unable to cooperate to complete the study.

### Treatment methods

Infectious mononucleosis children in the two groups were given symptomatic and supportive treatment, such as correction of water-electrolyte disturbances and acid-base imbalance, nutritional support treatment and antipyretic treatment for children with a high fever. On the basis of the above-mentioned treatments, the control group was additionally given ganciclovir (10 mg/kg) by intravenous infusion, once a day, seven days as a course of treatment, with a total of two courses. On the basis of the treatment in the control group, the study group was further treated with the combination of recombinant human interferon-α1b by nebulization inhalation, five μg/time, twice a day, with the treatment lasted for one week. *Both two groups were followed-up for three months*

### Observation indexes:

- ***Improvement of clinical symptoms***: the improvement time of clinical symptoms of IM children in the two groups were separately recorded, including the time for recovery of normal body temperature, time for disappearance of angina, time for disappearance of lymphadenopathy and time for disappearance of hepatosplenomegaly;
- ***Inflammatory indexes:*** peripheral venous blood samples (five ml) were collected from all children in the morning before and after treatment, and the levels of tumor necrosis factor-a (TNF-a), interleukin-6 (IL-6) and other inflammatory cytokines were detected by enzyme-linked immunosorbent assay (ELISA);
- ***Analysis of immune function condition:*** fasting blood samples were collected in all cases under fasting condition in the morning before and after treatment, and the levels of T-lymphocyte subsets by flow cytometry, including CD3^+^, CD4^+^, CD8^+^ and CD4^+^/CD8^+^, and the differences between the two groups before and after treatment were compared;

Comparative analysis of adverse drug reactions in two groups: incidence of adverse reactions were recorded, including nausea, diarrhea and other gastrointestinal reactions, rash, thrombocytopenia.

### Statistical analysis

All data were analyzed by SPSS 20.0 software. Measurement data were expressed as (*χ̅*±*S*). Two-independent-sample t-test was used for inter-group data analysis, paired t-test was applied to intra-group data analysis, and χ^2^ test was used for comparisons regarding percentages. *P*< 0.05 was considered statistically significant.

## RESULTS

After treatment, the time for body temperature dropped to normal, the time for recovery from cervical lymphadenopathy, the time for recovery from hepatosplenomegaly and the time for disappearance of angina and oral mucosal congestion in the study group were significantly shorter than those in the control group (*p=* 0.00) (Table-II).

**Table-II:** Comparative analysis of recovery time (d) of clinical symptoms after treatment in the two groups (n = 60 per group) (*χ̅*±*S*).

Grouping	Angina and oral mucosal congestion	Hepatosplenomegaly	Cervical lymphadenopathy	Body temperature recovery
Study group	6.34 ± 2.05	6.43 ± 1.38	6.55 ± 2.41	4.41 ± 1.04
Control group	9.70 ± 2.33	8.53 ± 2.16	8.73 ± 3.07	6.28 ± 1.75
*t*	8.40	6.62	4.33	7.12
*P*	0.00	0.00	0.00	0.00

The changes in inflammatory cytokines in the two groups before and after treatment are shown in Table-III, and the results indicated that TNF-a, IL-6 and other inflammatory cytokines in the two groups were significantly higher before treatment, but without significant difference between the two groups (*p>* 0.05). After treatment, levels of the above-mentioned indexes were decreased, and there were significant differences in the levels of inflammatory cytokines between the two groups (*p<* 0.05). After treatment, levels of TNF-a and IL-6 in the study group were significantly lower than those in the control group.

**Table-III:** Comparative analysis of changes in inflammatory cytokines in the two groups before and after treatment (n = 60 per group) (*χ̅*±*S*).

Grouping		Before treatment*	After treatment	t	P
TNF-ɑ (pg/mL)	Study group D	53.80 ± 21.33	3.07 ± 3.00	17.95	0.00
	Control group D	57.50 ± 20.10	7.19 ± 3.07	19.30	0.00
	*t*	0.98	7.44		
	*p*	0.33	0.00		
IL-6 (pg/mL)	Study group D	66.54 ± 53.62	3.18 ± 2.48	9.19	0.00
	Control group D	59.02 ± 48.28	6.45 ± 2.56	8.41	0.00
	*t*	0.81	7.11		
	*p*	0.421	0.00		

* p> 0.05, Dp< 0.05.

There was no significant difference in the levels of CD3^+^, CD4^+^, CD8^+^ and CD4^+^/CD8^+^ between the two groups before treatment (*p>* 0.05). After treatment, the indexes of CD3^+^ and CD8^+^ in the study group were significantly lower than those in the control group, and the levels of CD4^+^ and CD4^+^/CD8^+^ were significantly higher in the study group than those in the control group (Table-IV). After treatment, there was no significant difference in the incidence of drug adverse reactions between the study group and the control group (16.7% vs. 15%, *p=* 0.80) (Table-V).

**Table-IV:** Comparative analysis of T-lymphocyte subsets before and after treatment in the two groups (n = 60 per group) (*χ̅*±*S*).

Indexes		Study groupD	Control groupD	t	P
CD3^+^ (%)	Before treatment*	81.71 ± 9.70	81.45 ± 9.83	0.15	0.89
	After treatmentD	63.41 ± 4.51	69.28 ± 3.71	7.79	0.00
	*t*	15.03	8.54		
	*P*	0.00	0.00		
CD4^+^ (%)	Before treatment*	15.64 ± 5.22	14.82 ± 5.19	0.87	0.39
	After treatmentD	38.41 ± 3.73	30.84 ± 2.66	12.80	0.00
	*t*	28.20	22.80		
	*P*	0.00	0.00		
CD8^+^ (%)	Before treatment*	60.54 ± 10.67	63.83 ± 12.02	1.58	0.12
	After treatmentD	25.86 ± 4.71	29.11 ± 4.20	3.98	0.00
	*t*	24.73	20.54		
	*P*	0.00	0.00		
CD4^+^/CD8^+^	Before treatment*	0.27 ± 0.11	0.26 ± 0.14	0.57	0.57
	After treatmentD	1.35 ± 0.25	1.24 ± 0.31	2.09	0.04
	*t*	32.66	21.87		
	*P*	0.00	0.00		

* p> 0.05, Dp< 0.05.

**Table-V:** Comparative analysis of the incidence of drug adverse reactions between the two groups.

Groping	Rash	Nausea	Diarrhea	Thrombocytopenia	Incidence
Study group	3	2	2	3	10 (16.7%)
Control group	2	4	1	2	9 (15%)
c^2^					0.06
*P*					0.80

## DISCUSSION

Our results showed that after treatment, the clinical symptoms of the study group were significantly improved compared with the control group, specifically, the time for body temperature dropping to normal, the time for recovery from cervical lymphadenopathy, the time for recovery from hepatosplenomegaly and the time for disappearance of angina and oral mucosal congestion in the study group were significantly shorter than those in the control group after treatment, the levels of TNF-a, IL-6 and other inflammatory cytokines in the study group were significantly lower than those in the control group; and the indexes of CD3^+^ and CD8^+^ of T-lymphocyte subsets in the study group were significantly lower than those in the control group; after treatment, the levels of CD4^+^, CD4^+^/CD8^+^ in the study group were significantly higher than those in the control group. There was no significant difference in the incidence of drug adverse reactions between the study group (16.7%) and the control group (15%).

Ganciclovir, a broad-spectrum antiviral drug^10^, was characterized in that ganciclovir could block the synthesis of viral DNA strand by mainly inhibiting the activity of DNA polymerase and reverse transcriptase, with inhibitory action on the replication and proliferation of the virus. Moreover, ganciclovir was widely used in clinical practice with the advantages of fast effect-taking speed and long-lasting action time.^11^ Stockmann et al.^12^ considered that, among the antiviral drugs discovered so far, ganciclovir showed the strongest inhibitory effect on virus activity. The main action mechanism may be that ganciclovir could be transformed into active ganciclovir triphosphate by deoxyguanosine kinase in virus-infected cells, thus resulting in competitive inhibition on the binding of deoxyguanosine triphosphate and viral DNA polymerase, terminating the extension of viral DNA strand. Interestingly, the inhibition effect was found to be more obvious in virus-infected cells, thus playing a strong antiviral effect. A relevant study^13^ suggested that EBV, a member of the DNA virus family, had a strong affinity for lymphoid tissues. The immune system could be activated, and then cellular immunity and humoral immunity of the body were affected after acute infection of EBV, thereby increasing the total number of white blood cells and inflammatory mediators. However, it has been reported that a variety of cytokines and pathogenesis were involved in the occurrence and development of IM and nasopharyngeal carcinoma(NPC).^14^ Therefore, the single-target drug showed an unsatisfactory treatment effect. Similarly, the study of Pagano et al.^15^ found that the effect of a single antiviral drug in the treatment of IM was very limited.

The solution of symptomatic IM was not determined by controlling viremia, but by changing the host’s response to EBV infection.^16^ Interferon was an important immune factor secreted by the body after being attacked by a virus, with immune enhancement and anti-inflammatory medium function, wherein the recombinant human interferon-α, a tissue engineering product, had a broad-spectrum antiviral effect. Borsini et al.^17^ demonstrated that interferon played an important role against EBV infection. Interferon had a very obvious anti-EBV infection effect in vivo, but not obvious in vitro, and the reaction could instantaneously inhibit virus replication and weaken the amplification of CD8^+^ T-lymphocytes without the need to control primary infection.^18^ Barrat et al.^19^ suggested that interferon had the ability of inducing “interferon epigenomic characteristics” by activating potential enhancers and “labeling” chromatin, with certain beneficial effects on anti-virus response, antigen presentation, autoimmune and inflammatory molecules. As reported by a previous study, peripheral blood lymphocyte subsets were closely related to the condition of IM children,^20^ and functional polymorphism in immune response was an important factor leading to significant individual differences in susceptibility and prognosis of infectious diseases. Importantly, acute disease response was presented as a set of stereotyped disease manifestations which were mediated by proinflammatory cytokines and induced by many different pathogens. Functional polymorphisms of key cytokine genes with key roles in early immune response, including tumor necrosis factor-α (TNF-α), interleukin-6 (IL-6), interleukin-10 (IL-10) and interferon-γ (IFN-γ), were revealed to have important impacts on the severity and duration of disease and disease outcome after acute EBV infection.^21^ It has been suggested that interferon could reduce levels of IL-6 and TNF-α by regulating the action of pro-inflammatory cytokines induced by CD4^+^ and CD8^+^ T-lymphocytes^22^, and played an anti-inflammatory role in IM.

### Limitations

It includes small sample size, short follow-up time and the failure of including the blood immunoglobulin content of child patients, as well as transaminase and other liver dysfunction indicators in this study. In the further, with further increase of sample size, extension of follow-up time and continuous improvement of clinical work, the above-mentioned indicators would be studied to further confirm the effect of the treatment plan on humoral immunity and liver dysfunction of child patients.

## CONCLUSION

Through receiving ganciclovir combined with recombinant human interferon-α, rapid improvements of clinical symptoms, significantly decreased inflammatory cytokines, significantly improved T-lymphocyte function and no significant increase in adverse drug reactions were found in children with IM. Therefore, ganciclovir combined with recombinant human interferon-α may be safe and effective in the treatment of IM in children.

### Authors’ Contributions:

**LS and HS** designed this study, prepared this manuscript, are responsible and accountable for the accuracy and integrity of the work.

**JB and WZ** collected and analyzed clinical data.

**MW** significantly revised this manuscript.
